# Effectiveness of interventions utilising telephone follow up in reducing hospital readmission within 30 days for individuals with chronic disease: a systematic review

**DOI:** 10.1186/s12913-016-1650-9

**Published:** 2016-08-18

**Authors:** Amanda Jayakody, Jamie Bryant, Mariko Carey, Breanne Hobden, Natalie Dodd, Robert Sanson-Fisher

**Affiliations:** Health Behaviour Research Group, Priority Research Centre for Health Behaviour, University of Newcastle, HMRI Building, Callaghan, NSW 2308 Australia

**Keywords:** Patient readmission, Telephone follow up, Chronic disease

## Abstract

**Background:**

Rates of readmission to hospital within 30 days are highest amongst those with chronic diseases. Effective interventions to reduce unplanned readmissions are needed. Providing support to patients with chronic disease via telephone may help prevent unnecessary readmission. This systematic review aimed to determine the methodological quality and effectiveness of interventions utilising telephone follow up (TFU) alone or in combination with other components in reducing readmission within 30 days amongst patients with cardiovascular disease, chronic respiratory disease and diabetes.

**Methods:**

A systematic search of MEDLINE, the Cochrane Library and EMBASE were conducted for articles published from database inception to 19^th^ May 2015. Interventions which included TFU alone, or in combination with other components, amongst patients with chronic disease, reported 30 day readmission outcomes and met Effective Practice and Organisation of Care design criteria were included. The titles and abstracts of all identified articles were initially assessed for relevance and rejected on initial screening by one author. Full text articles were assessed against inclusion criteria by two authors with discrepancies resolved through discussion.

**Results:**

Ten studies were identified, of which five were effective in reducing readmissions within 30 days. Overall, the methodological quality of included studies was poor. All identified studies combined TFU with other intervention components. Interventions that were effective included three studies which provided TFU in addition to pre-discharge support; and two studies which provided TFU with both pre- and post-discharge support which included education, discharge planning, physical therapy and dietary consults, medication assessment, home visits and a resident curriculum. There was no evidence that TFU and telemedicine or TFU and post-discharge interventions was effective, however, only one to two studies examined each of these types of interventions.

**Conclusions:**

Evidence is inconclusive for the effectiveness of interventions utilising TFU alone or in combination with other components in reducing readmissions within 30 days in patients with chronic disease. High methodological quality studies examining the effectiveness of TFU in a standardised way are needed. There is also potential importance in focusing interventions on enhancing provider skills in patient education, transitional care and conducting TFU.

**Electronic supplementary material:**

The online version of this article (doi:10.1186/s12913-016-1650-9) contains supplementary material, which is available to authorized users.

## Background

Readmissions to hospital within 30 days of discharge are generally considered an unplanned or potentially avoidable event [1, 2]. In the United States (US), 1 in 5 Medicare fee-for-service patients are readmitted to hospital within 30 days of discharge and it is estimated that up to 90 % of readmissions within 30 days are unplanned [3]. Reported estimations of annual health system costs due to readmission range from $12 billion to $17.4 billion in the US [3, 4] and £1.6 billion in the United Kingdom (UK) [5]. Readmissions are also associated with human costs such as feelings of frustration and time lost from an individual’s usual role within the workplace and family [6].

Readmissions are highest amongst those with chronic diseases, in particular amongst patients with cardiovascular disease, chronic respiratory disease and diabetes [3, 7–10]. Patients with chronic heart failure have been reported to be at the highest risk of readmission to hospital within 30 days [7, 8, 11] with reported rates of 26.9 % amongst Medicare fee-for-service patients [3]. Individuals with chronic obstructive pulmonary disease (COPD) and diabetes also have high reported readmission rates (22.6–20 % respectively [3]) [7, 8, 12]. Patients with chronic disease discharged from hospital often have complex health care needs and treatment plans, which means the early discharge period is a challenging time for the patient and their carer [9, 13].

Inadequate discharge planning, poor follow up from community health care services, and a lack of patient and carer education in chronic disease self-management skills are believed to contribute to unplanned readmission [14, 15]. Healthcare guidelines in the UK and the US penalise hospitals by restricting government payments for excess unplanned readmissions within 30 days of discharge, based on the rationale that readmissions result from suboptimal care and are preventable [4, 16–18]. This has led to increased motivation to find effective strategies to reduce unplanned readmissions [1, 14, 19].

The effectiveness of a number of intervention strategies, including discharge planning, patient education, telephone follow up (TFU), home visits, and transition coaching, have been explored to reduce readmissions. Research to date has found no consistent evidence of a singular or multicomponent intervention in reducing readmission [14]. However previous systematic reviews have highlighted that TFU is a common component of successful randomised trials of multi-component interventions in reducing readmissions [14, 20]. Therefore it is a potentially promising intervention amongst patients with chronic disease. TFU, where a hospital or community health worker calls a recently discharged patient at home, is used to provide ongoing education, management of symptoms and prescribed medication, recognition of complications and reassurance to patients with the aim of facilitating a smooth transition into community or specialist health care [21, 22]. TFU is considered easy to implement and low cost [2, 21]. Telephone contact has been linked to increased patient satisfaction [23].

Several reviews to date have examined the effectiveness of TFU [2, 14, 21, 24]. Hansen and colleagues examined the effectiveness of 43 studies which used different types of singular and multi-component interventions in reducing 30 day readmissions in both surgical and medical patients [14]. Following assessment of included studies against Cochrane Effective Practice and Organisation of Care (EPOC) criteria, they found most were observational studies and there was extensive heterogeneity in content and context. They concluded there was no intervention, including TFU, which was consistently effective in reducing readmissions [14]. A Cochrane systematic review examined the effectiveness of TFU delivered by hospital-based staff on health outcomes in 33 studies involving 5110 surgical and medical patients [21]. While the main focus of the review was on psychosocial and physical outcomes, four studies reporting readmission outcomes amongst patients with cardiac disease were pooled together and no effect was found at three months. Again applying EPOC criteria, they found studies were of low methodological quality. Readmission outcomes at 30 days were not assessed. Another review by Bahr and colleagues focused on hospital based TFU as a singular intervention amongst medical and surgical patients, with no impact on readmissions within 30 days [2]. However they included descriptive studies and no formal assessment of methodological quality was performed. Crocker and colleagues in their review of three included studies also concluded that TFU alone is ineffective in reducing readmissions amongst general medical patients [24]. Risk of bias in study design was assessed but no formal scoring was reported. They did not assess 30 day outcomes and focussed solely on TFU delivered by a primary care team member, and therefore the results are not generalizable to more common hospital based models of TFU where calls are made by the discharge nurse.

While overall, these reviews suggest that the evidence for TFU in reducing readmissions is inconclusive, none have focussed specifically on hospitalised chronic disease patients, and therefore it is unclear to which results are generalizable to this population. Given the increasing prevalence and healthcare burden of chronic diseases, its disease complexity, and the development of government chronic disease strategies [25, 26], it is pertinent to examine the effectiveness of TFU in patients with one or more chronic disease separately from general medical and surgical patients. Therefore, the aim of this review is to assess the methodological quality and effectiveness of interventions using TFU in reducing readmission within 30 days amongst patients with cardiovascular disease, chronic respiratory disease and diabetes.

## Methods

### Data sources and searches

A systematic search of the MEDLINE, Cochrane Library, and EMBASE electronic databases was conducted from database inception to 19^th^ May 2015. A medical librarian was consulted to develop Medical Subject Heading (MeSH) search terms and keywords under three main groups: hospital readmission, TFU and chronic diseases (see Additional file 1 for search strategies for each database). The search was limited to papers published in English and human studies. Previous reviews of relevant literature and the reference lists of retrieved articles were manually searched to identify additional relevant papers.

### Study selection

Studies were included if: (1) they tested the effectiveness of TFU, either on its own or in combination with other intervention components. TFU was defined as a telephone call to the chronic disease patient initiated by the health provider post-discharge; (2) they met the EPOC criteria for study design [27], i.e., randomized controlled trials (RCTs), non-randomized controlled trials (NRCT), controlled before and after studies (CBA) with adjustment for confounders or interrupted time series designs (ITS); (3) it had a primary objective to reduce hospital readmissions within 30 days amongst individuals with one or more of the following chronic diseases: cardiovascular disease (such as heart disease and stroke), chronic respiratory disease (such as COPD or asthma) and diabetes (types 1 or 2); and (4) readmission was clearly defined and measured as readmission to hospital within 30 days of discharge. Studies which included patients with diabetes, cardiac or respiratory diseases as well as other diseases/ conditions were included if: a) the results were reported separately for the chronic diseases of interest to the present review; or b) patients with diabetes, respiratory or cardiac disease comprised 75 % or more of the sample. Studies were excluded if they: (1) only offered a hotline or a 24 h telephone service that allowed a patient to initiate contact with a health provider, or telemedicine interventions where the patient only answered pre-recorded questions over the telephone without any additional TFU; (2) did not report readmission outcomes separately from other outcomes such as mortality; or (3) examined readmissions in paediatric, obstetric, or psychiatric populations.

### Data extraction and quality assessment

The titles and abstracts of all papers identified in the literature search were initially assessed for relevance and rejected on initial screening if the reviewer (AJ) could determine that the study did not meet inclusion criteria. If an article did not clearly indicate whether inclusion criteria were met, the article was retained for full-text review. Full text versions of the remaining papers were assessed against the inclusion criteria separately by two authors (AJ and JB) with discrepancies resolved through discussion. Studies which met all criteria were retained for inclusion in the review (Additional file 2). Included studies were assessed separately by two of four authors (AJ, BH, ND, MC) against the EPOC risk of bias methodological criteria [27]. The nine standard criteria examined whether allocation sequence was adequately generated and adequately concealed, whether baseline outcome measurements or characteristics were similar, whether incomplete outcome data was adequately addressed, whether knowledge of the allocated interventions was adequately prevented during the study, whether the study adequately protected against contamination, whether the study was free from selective outcome reporting or from other risks of bias [27]. Discrepancies were resolved by discussion between the authors.

### Data synthesis and analysis

To assess intervention effectiveness, the following data was extracted from each study which met the inclusion criteria: (1) sample characteristics, (2) type of intervention and comparison group, (3) outcomes and measures, and (4) main findings regarding readmissions within 30 days. The included studies’ intervention components were organised around the pre-discharge and post-discharge periods. In order to reflect these periods, components were classified into five intervention categories for narrative synthesis.

## Results

### Search results

**Fig. 1:**
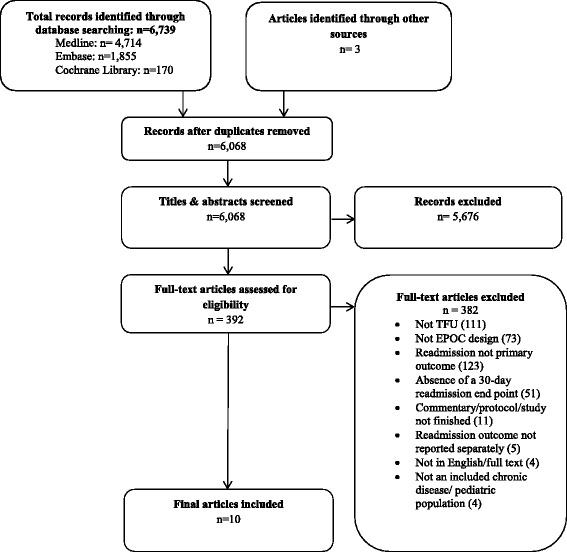
Flow diagram of search strategy and selection

A total of 6,739 articles were identified based on the specified search strategy. After removal of duplicates and assessment against eligibility criteria, ten articles met criteria for inclusion in the review. A flow chart of the literature search and paper identification is provided in Fig. 1.

### Characteristics of included studies

Additional file 2 summarises the characteristics of included studies. Only one study was identified as a RCT [28], eight were NRCTs [29–36], and one was a CBA study [37]. Most studies were conducted with patients with heart failure (*n =* 7) [29, 31–36], two studies involved patients with COPD [28, 30], and one study included stroke patients [37]. No included studies targeted patients with diabetes. Studies were conducted in : US [29, 31, 34–36], Taiwan [37], Ireland [33], Denmark [28, 30], and The Netherlands [32]. Two studies examined readmission across multiple hospital sites [28, 31], whilst the remaining examined readmission within a single hospital site. Study sample sizes ranged from 70 [33] to 375 [34].

#### Intervention delivery

TFU was provided by nurses in seven of the ten studies [28–32, 36, 37], and by resident doctors in one study [34]. One study used trained volunteers who were university students pursuing a premedical track [35], and one study did not report who made the follow up call [33].

#### Intervention intensity and content

Varying numbers of telephone calls were provided as part of follow-up, ranging from one [29, 32, 34] up to approximately 16 [31]. Seven studies reported the content of the TFU calls, however the level of detail reported varied [29, 31, 32, 34–37]. Four studies reported TFU which included an assessment of the patient’s health and adherence to treatment, for example, symptom control, medication compliance, dietary adherence, care-management orders, and activity capacity [29, 31, 35, 37]. Two studies reported TFU which included education or coaching for the patient regarding self-care skills or what to do if they are not feeling well [31, 35]. Two studies monitored patients’ health [32, 36], and the nurse intervened as necessary [36] but provided no further detail of the call content. Record and colleagues used TFU to assess the patient’s experience of the care transition and their understanding of the hospital stay [34]. Appointments were made during TFU for follow up care in two studies [32, 34]. Dai and colleagues also asked the patient about any readmissions they had since discharge [37]. Three of the studies which provided information on the content of the call, reported using a structured format which followed a set of questions or a script [29, 31, 35].

#### Outcome measurements

Two studies measured unplanned readmission [33, 37] whilst the remaining studies did not distinguish between planned or unplanned readmissions. Four studies reported a chronic disease specific readmission outcome [29, 33, 35, 36], one study reported all-cause readmissions [37], four studies reported both all-cause and chronic disease readmission outcomes [28, 30–32], and one study did not specify the cause of readmission [34].

### Methodological quality of included studies

The methodological quality of included studies is shown in Table 1. One study was rated as low methodological risk on eight of the nine criteria [28], however most studies scored low risk on only two to five of the nine criteria [29–37]. Studies performed poorest on generation of allocation sequence, allocation concealment, outcome and baseline similarities and all but one study [34] scored a high risk for contamination.

**Table 1:** Methodological quality of included studies assessed by the EPOC risk of bias criteria

Study	Design	Allocation sequence	Allocation concealment	Baseline outcome	Baseline characteristics	Incomplete outcomes	Blinding	Contamination	Selective reporting	Other bias
Anderson, 2005 [29]	NRCT	H	H	U	H	U	L	H	L	U
Dai, 2003 [37]	CBA	H	H	U	H	L	U	H	L	H
Jaarsma, 1999 [32]	NRCT	U	U	L	L	U	L	H	L	L
McDonald, 2001 [33]	NRCT	U	U	U	L	L	L	H	L	L
Naylor, 1994 [36]	NRCT	U	U	U	H	U	L	H	L	L
Record, 2011 [34]	NRCT	H	U	L	H	U	L	U	L	L
Riegel, 2006 [31]	NRCT	U	L	U	U	L	L	H	L	L
Sales, 2014 [35]	NRCT	U	U	U	L	L	L	H	L	L
Sorknaes, 2011 [30]	NRCT	H	H	U	L	L	L	H	L	L
Sorknaes, 2013 [28]	RCT	L	L	L	L	L	L	H	L	L

Studies coded as high risk are labelled with “H”, those coded as low risk are labelled with an “L” and those studies coded as unclear (which did not provide sufficient information to assess risk of bias) are labelled with “U”

### Effectiveness of interventions in reducing readmission

Table 2 presents the included studies by effectiveness and intervention category. No included studies tested the effectiveness of TFU as a singular intervention.

**Table 2:** Categories of interventions by effectiveness

Intervention categories	References	
	Effective	Not effective
Telephone follow-up and pre-discharge interventions		
• Pre-discharge patient education and telephone follow-up	Sales et al. [35]	-
• Pre-discharge planning and telephone follow-up	Dai et al.-craniotomy [37]; Naylor et al.-medical [36]	Dai et al.-stroke [37]; Naylor et al.-surgical [36]
Telephone follow-up and post-discharge interventions		
• Telephone follow-up, printed education materials and case management	-	Riegel et al. [31]
Telephone follow-up and pre-and post-discharge interventions		
• Pre-discharge education, telephone follow-up, and home visits	-	Jaarsma et al. [32]
• Pre-discharge education, physical therapy and dietary consult, discharge planning, telephone follow up and home visits	Anderson et al. [29]	-
• Patient-centred, transition-focused care curriculum for residents, medication assessment, telephone follow up and home visits	Record et al. [34]	-
• Pre-discharge education, dietetic consults, telephone follow-up, and primary care or specialist follow-up	-	McDonald et al. [33]
Telephone follow-up and telemedicine		
• Telephone follow-up, telemedicine and telephone hotline	-	Sorknaes et al. 2011 [30]; Sorknaes et al. 2013 [28]

#### TFU and pre-discharge interventions

Three studies evaluated pre-discharge education or discharge planning interventions in addition to TFU, with mixed results [35–37]. The first study, a NRCT amongst patients with congestive heart failure, used trained volunteers to provide patient education, and medication instructions pre-discharge. This was followed by four structured telephone calls post-discharge reiterating discharge instructions and coaching about when to call primary care physician if not feeling well [35]. Compared with standard care, the intervention group had lower rates of 30-day readmissions (7 % vs 19 %; *P <* .05). The second study, a NRCT amongst elderly cardiac patients, involved specialist nurses providing an individualised discharge planning protocol and a minimum of two telephone follow up calls by the nurse within two weeks of discharge [36]. The discharge planning included ongoing assessment, development of a discharge plan with the patient and health care team, education, coordination and interdisciplinary communication. Readmissions within two weeks were reduced in the medical intervention group compared with the control group (4 %; 16 % *P <* 0.02), but there was no significant difference between surgical intervention and control groups (7 %;11 %). Dai and colleagues reported on a CBA study amongst stroke and craniotomy patients [37]. Intervention participants received discharge planning, including a needs assessment, pre-discharge instruction, health care coordination and referrals, followed by TFU conducted by a nurse over two sessions. Unplanned readmission was significantly reduced in the craniotomy intervention group (5.4 %) compared to the control group (17.8 %; *P =* 0.04) at one month follow‐up, but not among stroke patients (1 % intervention compared to 4.2 % control; *P =* 0.31).

#### TFU and other post-discharge interventions

Riegel and colleagues examined the effectiveness of a heart failure TFU case management intervention delivered by nurses combined with the provision of post-discharge printed education pamphlets and consultation with physicians in community hospitals on the US-Mexico border [31]. Nurses used a decision support software program when telephoning intervention patients and conducted a mean of 10.5 calls per patient starting 5 days post-discharge. The program provided guidance to the nurse about decisions related to patient medication adherence, diet, signs and symptoms of worsening illness, and determined the frequency of calls. There was no effect of the intervention on all-cause (8.7 % vs 13.8 %; *P =* 0.42) and heart failure readmissions at one month post-discharge (15.9 % vs 20.0 %; *P =* 0.65).

#### TFU and pre-and post-discharge interventions

Four studies evaluated TFU with both pre- and post-discharge components with heart failure patients, with mixed evidence of effectiveness [29, 32–34]. Record and colleagues compared standard care to a physician-led intervention which incorporated a patient-centred, transition focused care curriculum for resident doctors at one teaching hospital in the US [34]. The trained doctors provided patients with a medication review, a call to their “outpatient provider”, a home visit and one TFU call to assess the patients’ experience of transition care and plans for follow up. The exact timing and length of the TFU call was not reported. The probability of survival 30 days post-discharge, without readmission for heart failure, was higher for the intervention group (*P =* .046). Anderson compared standard care for patients to a nurse case manager-delivered intervention comprising of pre-discharge education, physical therapy and dietary consultations, discharge planning, one TFU call and 6–20 home visits [29]. TFU, conducted within two weeks of discharge, involved assessment of symptom control, medication compliance, dietary adherence, and activity capacity. Readmission within 30 days was reduced significantly in the intervention group compared with standard care (I = 6.0 % vs. C = 22.1 %; *P =* 0.01). Jaarsma and colleagues tested a comprehensive intervention which included inpatient education, dietary and physical therapy consults, discharge planning and home visits with TFU by nurses within one to two weeks post-discharge [32]. No effect on reducing readmissions within 30 days of discharge was observed. A fourth small study (*n =* 70) tested an intervention involving inpatient education with the patient’s carer, dietetic consults, follow up appointments in an outpatient clinic, and TFU calls three days post-discharge and weekly thereafter [33]. No differences between the groups were observed, with both groups having a zero rate of unplanned admissions within 30 days.

#### TFU and information technologies for monitoring patients by distance (telemedicine)

Sorknaes and colleagues conducted two separate studies to examine the effectiveness of a daily teleconsultation by video with a nurse for five to nine days after discharge amongst COPD patients compared to patients receiving usual care [28, 30]. Nurses made clinical observations, measured oxygen saturation levels and lung function, and informed patients how to prevent exacerbations and how to use their medication. The nurses made one TFU call one week after the teleconsultations however no call detail was reported. Neither study reported a significant difference in mean total readmissions or COPD readmissions between intervention and control groups.

## Discussion

This systematic review examined the effectiveness of TFU in reducing readmission within 30 days of discharge among patients with cardiovascular disease, chronic respiratory disease and diabetes. Of the ten intervention studies which met the EPOC research design criteria, five were effective in reducing readmissions within 30 days. However the methodological quality of studies was poor. Apart from one low risk study, most had similar limitations, which weakens the overall strength of evidence. There was a lack of uniformity in how readmission was measured which highlights the need for consistency and precision in the measurements used in studies aiming to reduce readmission. Most studies identified were single site interventions and thus findings may have limited generalisability. In addition, the studies presented wide variation in standard care provided to control groups. Some studies included very little information on what constituted standard care. This made it difficult to interpret study results in relation to the circumstances under which the interventions were likely to be effective or ineffective.

All identified studies combined TFU with other intervention components. All three studies evaluating TFU with pre-discharge interventions showed effectiveness, however in two studies readmission was significantly reduced in only one of the two intervention groups, i.e. in the craniotomy group and not the stroke group [37]; and in the medical group and not the surgical group [36]. Two of four studies evaluating TFU with both pre- and post-discharge components were effective [29, 34]. There was no evidence that TFU and telemedicine or TFU and post-discharge interventions was effective, however, only one to two studies examined each of these types of interventions. On balance, the evidence for TFU is equivocal. There is some suggestion however that combining TFU with pre-discharge intervention components may be promising but further interventions are needed to confirm whether this is the case for both medical and surgical patients with chronic disease. Although the effective studies all offered some form of continuity or bridging for the patient from the hospital to the community setting, none included components distinctive from the ineffective studies. This equivocal finding aligns with that of Hansen and colleagues, who also found no conclusive evidence for a multi-component intervention in reliably reducing readmissions amongst general and surgical patients [14].

Questions also still remain as to whether TFU itself is the effective component or not. The outcomes of TFU may be masked by many factors such as individual professional and patient actions and behaviour, social interactions and environmental settings [21]. Further randomised trials of high methodological quality examining the effectiveness of TFU in a standardised way are needed. In particular, given the lack of detail given in many included studies with regards to TFU, it may be warranted to examine the intensity, content and length of calls needed to achieve a significant effect for such patients. TFU is a popular feature of interventions in reducing readmissions, however given limited health resources, the specific details surrounding the effectiveness of TFU for patients with chronic disease still needs to be tested.

Seven of the ten included studies focused on patients with heart failure. Although chronic diseases share common features in terms of intermittent exacerbation of disease, persistence over time and are rarely cured [38], there are differences with respect to the type and intensity of treatment, symptoms and the professional care needed. Therefore, study results derived from one chronic disease population cannot necessarily be generalised to other chronic disease groups. Given this, there is a need for more intervention research on reducing 30 day readmissions for patients with other prevalent chronic disease such as diabetes and chronic respiratory disease.

Patient-centred care requires communication between hospital and community based physicians; ensuring patients do not experience a gap in care and understanding. The roles of these health professionals are critical to preventing readmission [39]. One included study focused on training hospital doctors in patient-centred transitional care through telephoning community physicians, home visits to the patient and conducting TFU which resulted in a significant reduction in 30 day readmissions [34]. However, most studies focused on patient-level interventions rather than provider-level change. Record and colleague’s study points to the potential importance of enhancing provider skills in patient education, transitional care and conducting TFU calls.

This review had a number of limitations. Firstly, a meta-analysis was not possible due to the wide variation in interventions between studies and readmission measures used. Secondly, many included studies were of low methodological quality and lacked detail making it difficult to determine the content or effectiveness of the interventions and to draw firm conclusions applicable to other hospitals and communities. Lastly, it is acknowledged that data on rates of readmissions will inevitably include some readmissions which are appropriate and unavoidable, for example, when a readmission is medically necessary due to an unavoidable change in chronic condition [40, 41]. Although two of the included studies measured unplanned readmissions, no studies measured avoidable readmissions. This is mainly due to the fact there is no agreed method of measuring avoidable readmissions [40]. Therefore data on rates of readmissions included in this review may be overestimated in terms of true avoidability.

## Conclusions

Although there is increasing priority being placed on reducing readmissions within 30 days, the evidence for the effectiveness of TFU alone or in combination with other intervention components in reducing readmissions in patients with chronic disease remains inconclusive. However despite the equivocal findings, there remain important implications for practice. Due to a lack of studies, there is no well-controlled evidence to suggest that TFU in isolation is an effective strategy. TFU combined with pre-discharge interventions show some promise, however, results are not consistent across patient groups. This may suggest the importance of ensuring that the pre-discharge and / or TFU intervention components are carefully tailored to the needs of the patient group. There is also potential importance in focusing interventions on enhancing provider skills in patient education, transitional care and conducting TFU. In generating good research evidence in this area, priority should be given to conducting studies of high methodological quality. Where possible, studies should be multi-site in order to enhance generalisability, and measurements of readmission need to be consistent across studies. In order to build upon the existing evidence-base, there is merit in focussing research efforts on the evaluation of delivery of standardised TFU in combination with pre-discharge interventions.

## Additional files

Additional file 1: Database search strategies. A complete list of the search strategies conducted in Medline, Embase and the Cochrane library. (DOCX 22 kb) Additional file 2: Study characteristics of included studies. Extracted data from each included study is presented and is organised by: (1) sample characteristics, (2) type of intervention and comparison group, (3) outcomes and measures, and (4) main findings regarding readmissions within 30 days. (DOCX 38 kb)

## Abbreviations

CBA: Controlled before and after studies
COPD: Chronic obstructive pulmonary disease
EPOC: Cochrane Effective Practice and Organisation of Care
ITS: Interrupted time series designs
MeSH: Medical Subject Heading
NRCT: Non-randomized controlled trials
RCT: Randomized controlled trials
TFU: Telephone follow up
UK: United Kingdom
US: United States
