# Supplementary material for: Effectiveness of interventions utilising telephone follow up in reducing hospital readmission within 30 days for individuals with chronic disease: a systematic review
Source: BMC Health Serv Res. 2016 Aug 18;16:403. doi: 10.1186/s12913-016-1650-9 (PMC4990979; doi:10.1186/s12913-016-1650-9)
Supplement: Additional file 1: — Database search strategies. A complete list of the search strategies conducted in Medline, Embase and the Cochrane library. (DOCX 22 kb) [file 12913_2016_1650_MOESM1_ESM.docx]

**Additional File 1: Medline, Embase and Cochrane database search strategies.**

1. **Database: OVID MEDLINE 1946 to May 2015**

| **Search** | **Query** |
| --- | --- |
| #1 | (re-admission* or re-admit* or readmission* or readmit* or post discharge* or postdischarge* or re-hospitali* or rehospitali* or multiple admi* or multiple stay*).tw. |
| #2 | Patient Readmission/ OR Patient Discharge/ OR Hospitalization/ |
| #3 | 1 OR 2 |
| #5 | exp Telephone/ |
| #6 | telephone follow up.mp. |
| #7 | (telecommunication* or tele?communication* or electronic communication or telephon* or phone or phone call* or follow?up call* or follow up* or call?back or calls or calling or call or tele?health or tele?medicine or tele?nursing or tele* or home monitoring or home care services).tw. |
| #8 | 5 OR 6 OR 7 |
| #9 | Diabetes Mellitus, Type 2/ or Diabetes Mellitus/ or Diabetes Mellitus, Type 1/ |
| #10 | exp Respiratory Tract Diseases/ |
| #11 | Cardiovascular Diseases/ |
| #12 | exp Heart Diseases/ |
| #13 | exp Vascular Diseases/ |
| #14 | congestive heart failure.tw. |
| #15 | 9 or 10 or 11 or 12 or 13 or 14 |
| #16 | 3 AND 8 AND 15 |
| #17 | limit 16 to (english language and humans) |
| #18 | limit 19 to "all child (0 to 18 years)" |
| #19 | 17 not 18 |
| #20 | limit 19 to (case reports or comment or congresses or editorial or letter or news or newspaper article) |
| #21 | 19 not 20 |

1. **Database: OVID Embase Classic + Embase 1947 to May 2015**

| **Search** | **Query** |
| --- | --- |
| #1 | (re-admission* or re-admit* or readmission* or readmit* or post discharge* or postdischarge* or re-hospitali* or rehospitali* or multiple admi* or multiple stay*).tw. |
| #2 | Hospital readmission/ OR hospital discharge/ OR hospitaliszation/ |
| #3 | 1 OR 2 |
| #4 | telephone/ or telephone follow up.mp |
| #5 | (telecommunication* or tele?communication* or electronic communication or telephon* or phone or phone call* or follow?up call* or call?back or calls or calling or call or tele?health or tele?medicine or tele?nursing or tele* or home monitoring or home based services).tw. |
| #6 | 4 OR 5 |
| #7 | diabetes mellitus/ |
| #8 | insulin dependent diabetes mellitus/ |
| #9 | non insulin dependent diabetes mellitus/ |
| #10 | cardiovascular disease/ |
| #11 | exp heart disease/ |
| #12 | exp vascular disease/ |
| #13 | exp respiratory tract disease/ |
| #14 | congestive heart failure.tw. |
| #15 | 7 or 8 or 9 or 10 or 11 or 12 or 13 or 14 |
| #16 | 3 and 6 and 15 |
| #17 | limit 16 to (human and english language) |
| #18 | limit 17 to (conference abstract or editorial or letter) |
| #19 | 17 not 18 |

1. **Database: The Cochrane Library - from inception to May 2015**

| **Search** | **Query** |
| --- | --- |
| #1 | MeSH descriptor: [Patient Discharge] explode all trees |
| #2 | MeSH descriptor: [Patient Readmission] explode all trees |
| #3 | MeSH descriptor: [Hospitalization] explode all trees |
| #4 | #1 or #2 or #3 |
| #5 | (re-admission* or re-admit* or readmission* or readmit* or post discharge* or postdischarge* or re-hospitali* or rehospitali* or multiple admi* or multiple stay*) .tw. |
| #6 | 4 or 5 |
| #7 | MeSH descriptor: [Telephone] explode all trees |
| #8 | telephone follow up.mp. |
| #9 | (telecommunication* or tele?communication* or electronic communication or telephon* or phone or phone call* or follow?up call* or call?back or calls or calling or call or tele?health or tele?medicine or tele?nursing or home monitoring or home based services) .mp. |
| #10 | #7 or #8 or #9 |
| #11 | MeSH descriptor: [Diabetes Mellitus] explode all trees |
| #12 | MeSH descriptor: [Diabetes Mellitus, Type 1] explode all trees |
| #13 | MeSH descriptor: [Diabetes Mellitus, Type 2] explode all trees |
| #14 | MeSH descriptor: [Cardiovascular Diseases] explode all trees |
| #15 | MeSH descriptor: [Heart Diseases] explode all trees |
| #16 | MeSH descriptor: [Vascular Diseases] explode all trees |
| #17 | MeSH descriptor: [Respiratory Tract Diseases] explode all trees |
| #18 | congestive heart failure.tw. |
| #19 | 11 or 12 or 13 or 14 or 15 or 16 or 17 or 18 |
| #20 | 6 and 10 and 19 |
