# Supplementary material for: Effectiveness of interventions utilising telephone follow up in reducing hospital readmission within 30 days for individuals with chronic disease: a systematic review
Source: BMC Health Serv Res. 2016 Aug 18;16:403. doi: 10.1186/s12913-016-1650-9 (PMC4990979; doi:10.1186/s12913-016-1650-9)
Supplement: Additional file 2: — Study characteristics of included studies. Extracted data from each included study is presented and is organised by: (1) sample characteristics, (2) type of intervention and comparison group, (3) outcomes and measures, and (4) main findings regarding readmissions within 30 days. (DOCX 38 kb) [file 12913_2016_1650_MOESM2_ESM.docx]

| **Reference** | **Sample** | **Intervention** | **Control** | **Outcome measures and follow up** | **Readmission findings within 30 days** |
| --- | --- | --- | --- | --- | --- |
| **Anderson *et al.*, 2005^24^**  USA  NRCT | *N=* 121  Mean age: I:81; C:77  % Male: I:39; C:38  Diagnosis: CHF  Setting: Bridgeport Hospital, Connecticut.  Inclusion: CHF, symptoms consistent with CHF, and radiographic  evidence of CHF.  Exclusion: refusal to participate, myocardial infarction within  prior 12 weeks, unstable angina, confusion, planned discharge to extended care facility, > 5 significant comorbidities, renal failure requiring dialysis, impending cardiac surgery, or continued NYHA class IV CHF  despite maximal medical treatment. | *N=*44. “Clinical pathway program” incl. one-on-one individualised inpatient education by cardiac nurse educator, physical therapy evaluation, a dietary consult, d/c planning with coordinated home care and TFU.   - Number of calls: 1 - Conducted: within 2 weeks of d/c. - Mean length of call: 15 mins. - Person who made call: nurse case manager - Content: ten questions designed to assess symptom control, medication compliance, dietary adherence, and activity capacity. | *N=*77. Education from regular nursing staff; dietary and physical therapy consults at discretion of attending physician; TFU not routinely performed; home health care carried out but not by trained nurses. | Outcome measures:   - CHF readmission rate   Follow-up: 30 days and 6 months. | - Readmission was reduced in the intervention group at 30 days (I=6.0% vs. C=22.1%; p=0.01) |
| **Dai *et al.*,**  **2003^32^**  Taiwan  CBA | *N=*283.  Mean age (yrs): Craniotomy: 52.28; Stroke:64.96.  % Male: Craniotomy: 59; Stroke: 59.  Diagnosis: Stroke, Craniotomy.  Setting: Teaching hospital, Taiwan.  Inclusion: Stroke pts with: physical disability; could follow directions; had potential for functional improvement. Craniotomy pts with: physical disability; cognitive impairment; pressure sore, endotracheal/ tracheostomy or nasogastric tube; chronic disease or complication.  Exclusion: NR. | *N=*56 (craniotomy); *N=* 99 (stroke). Comprehensive pre d/c planning including early needs assessment; instruction; care co-ordination; referral for continuing care; procurement of medical devices or social resources.   - Number of calls: 2/pt. - Conducted: 1 week and at 1 month post d/c. - Mean length of call: NR. - Person who made call: Nurse. - Content: Obtain data on readmission. Responded to questions regarding health matters, self-care and other instruction as needed. | *N=*56 (craniotomy); *N=*72 (stroke). General routine care (no pre-d/c planning).   - Number of calls: 2/pt - Conducted: 1 week and 1 month post d/c. - Mean length of call: NR - Person who made call: Nurse. - Content: Obtain data on readmission. Instruction given only when pt or caregiver initiated questions. | Outcome Measures:   - Rate of all-cause unplanned readmission.   Follow-up: 1 month. | - Unplanned readmission was significantly reduced in the craniotomy I group compared to the C Group (3% vs 10%; p=0.04). - No significant difference in readmission was found between the 2 stroke groups (3% vs 1%; p=0.31 |
| **Jaarsma *et al.*, 1999^27^**  Netherlands  NRCT | *N=* 179.  Mean age (yrs): I:73±9 ; C:73±9.  % Male: 58.  Diagnosis: HF.  Setting: cardiology ward of the University Hospital in Maastricht.  Inclusion: (NYHA) functional class III and IV; a diagnosis of heart failure at least 3 months before admission, aged at least 50yrs and literate in Dutch.  Exclusion: co-existing, severe, chronic debilitating disease; resided in; or planned to be discharged to a nursing home; psychiatric diagnosis; had CABG; PTCA or valve replacement in the last 6 months or were expected to have such a treatment within 3 months; refused to give informed consent. | *N=*84. Pre d/c intensive, systematic and planned education by a study nurse about consequences of HF. Home visit post d/c to continue education.   - Number of calls: 1. - Conducted: 1 week post d/c. - Mean length of call: NR. - Person who made call: Nurse. - Content: assess potential problems and to make an appointment for a home visit. | *N=*95. Usual care. May have involved written or oral advice dependent on nurse or physician. | Outcome measures:   - All-cause and cardiac readmission rates and days   Follow up: 1, 3 and 9 months | - No significant differences noted for 1 month follow-up for either all-cause or cardiac readmissions rates and days. |
| **McDonald *et al.*, 2001^28^**  Ireland  NRCT | *N=*70  Mean age (yrs):I: 69.9±11.3; C: 67.9±12.0  % Male: I:71; C:63  Diagnosis: CHF  Setting: St. Vincent’s University Hospital, Ireland.  Inclusion: CHF if all 4 criteria present: history and examination compatible with CHF, chest X-ray appearance of congestion, echocardiography evidenced left ventricular dysfunction and response to initial therapy  Exclusion: Pts presenting  myocardial infarction or  unstable angina, or where failure was not primary problem and illnesses that could compromise  survival over duration of study or cognitive impairment. | *N=*35. Inpatient specialist nurse-led education and dietetic consults. Education programme on weight monitoring, disease and medication understanding and salt restriction. Advice given to the pt’s carer or next of kin. Pt discharged with physician referral letter. Attended clinic to check clinical status.   - Number. of calls: NR - Conducted: at 3 days and then weekly until 12 weeks. - Mean length of calls: NR - Person who made call: NR - Content: NR | *N=*35. Routine care. Normal primary physician follow-up. Physician told to inform study centre of any admissions. | Outcome measures:   - CHF unplanned readmission rate.   Follow up: 30 & 90 days | - 20% 30-day readmission rate prior to enrolment reduced to 0% following index admission in both I and C groups. |
| **Naylor *et al.*, 1994^31^**  USA  NRCT | *N=*276  *Mean age (yrs):*Medical: I: 76 ± 5.2; C: 76 ± 4.9. Surgical: I: 75 ± 4.4; C: 75± 4.3.  *% Male:* Medical: I: 57; C: 41. Surgical: I: 82; C:61.  Diagnosis: Medical: CHF and angina/MI. Surgical: coronary artery bypass graft and cardiac  valve replacement.  *Setting:* Hospital of the University of Pennsylvania  *Inclusion:* ≥70 yrs; speak English.  be alert and oriented and be able to be reached by telephone after discharge.  *Exclusion:* NR | *N=*72 (medical); *N=*68 (surgical). Pts received routine plan and comprehensive, individualized  discharge planning by gerontologic clinical nurse specialists: initial and ongoing assessment of pt needs; development of discharge plan with pts, physician, carer and health care team; education, coordination of discharge plan; interdisciplinary communication; evaluation; telephone hotline.   - Number of calls: min. 2 - Conducted: during 2 weeks post-discharge - Mean length of call: NR - Person who made call: nurse specialist - Content: to monitor patients progress and intervene when necessary. | *N=*70 (medical); *N*=66 (surgical). Pts received hospital’s routine discharge plan. Uncomplicated discharges managed by the pts physician and primary nurse. Discharges which needed  coordination of services and external providers, involved  social workers and community nursing coordinators. | Outcome measures:   - heart disease readmission rates   Follow up:  2, 6 and 12 weeks | - Medical: within 2 weeks of discharge: I: 3 (4%); C: 11 (16%); P<0.02; Difference = -12% (-22%--2%) - Surgical: within 2 weeks of discharge: I: 5 (7%); C: 7 (11%); NS; difference: -4% (-14%-6%) |
| **Record *et al.* et al., 2011^29^**  USA  NRCT | *N=*375.  Mean age (yrs): C:71.5  % Male: C:42.  Diagnosis: CHF  Setting: Johns Hopkins Bayview Medical Center.  Inclusion: NR  Exclusion: NR | *N=*52. Pt centered, transition-focused care curriculum for internal medicine residents. 1) medication adherence assessment; 2) telephone call(s) to outpatient provider(s). Visited selected pts at home or in nursing facility.   - Number of calls: 1 - Conducted: post-discharge - Mean length of call: NR - Person who made call: Intervention resident - Content: to assess pt’s experience of the care transition and his or her understanding of the hospital stay and plans for follow-up. | *N=* 323. Standard teaching team. | Outcome measures:   - Survival analysis without HF readmission   Follow-up: 30 days | - Probability of survival 30 days without readmission for HF was higher for the intervention team (P=.046). |
| **Riegel *et al.*, 2006^26^**  USA  NRCT | *N=*134  Mean age (yrs): I: 72.7±11.2; C: 71.6±10.8.  % Male: 46.3.  Diagnosis: HF.  Setting: Community hospitals close to the US-Mexico border.  Inclusion: Self-identified as Hispanic; diagnosis of HF; living in the community; planning to return to the community after hospital d/c.  Exclusion: History of cognitive impairment; renal insufficiency requiring dialysis; acute MI ≤ 30 days without history of CHF; serious or terminal condition; major and uncorrected hearing loss; no access to phone or informed consent. | *N=*69. Printed HF education materials in desired language mailed to pts monthly and as needed. Pts report given to physician at regular intervals. Telephone call conducted 5 days after discharge and as required thereafter.   - Number of calls: M=13.5 (SD 5.9; Med 13; IR 11–16) - Conducted: Within 5 days after discharge and a matter of judgement thereafter. - Mean length of call: NR. - Person who made call: Nurse case managers. - Content: Nurses guided by “At home with HF” software program, which covered medication adherence, diet, signs and symptoms of worsening illness. Nurse also provided support. | *N=*65. UC. Pts were educated regarding HF management before hospital discharge, assuming that someone bilingual was available to translate. | Outcome measures:   - HF readmission rates - All cause readmission.   Follow up: 1, 3 and 6 months. | - No significant difference between I and C in HF readmission rates at 1 mth (8.7% vs 13.8%; p=0.42). - No significant difference between I and C in all cause readmission rates at 1 mth (15.9% vs 20.0%; p=0.65). |
| **Sales *et al.*, 2014^30^**  USA  NRCT | *N=*137  Mean age (yrs): I: 72.5 6 14.8; C: 72.6 6 13.4  % Male: I: 37.1; C: 47.8  Diagnosis: CHF  Setting: New York Methodist Hospital  Inclusion: Cardiologist confirmed CHF as primary diagnosis.  Exclusion: Dementia or other severe psychiatric illness, and pts transferred to another hospital before d/c. | *N=*70. Trained volunteers gave education to pt before d/c. CHF education, medications review, review of non-pharmacological D/C instructions; and encourage importance of follow-up.   - Number of calls: 4 - Conducted: within 24 to 48 hours of d/c.; weekly for 1 month. - Mean length of call: 15 mins. - Person who made call: trained volunteer - Content: How are you doing?; Are you taking your medications as prescribed?; When is your follow-up appointment?; Are you weighing yourself daily?; Are you following a low-salt diet?; Are you exercising? Provided education & coaching. | *N=*67. Standard care. Standardized d/c instruction sheet; nurse-led review of medications and pt education. Pt provided with schedule appointments with their primary care physician. | Outcome measures:   - CHF Readmission   Follow up: 30 days | - I: Decreased 30-day readmissions (7% vs 19%; P <..05) with relative risk reduction of 63% and absolute risk reduction of 12%. |
| **Sorknaes *et al.*, 2011^25^**  Denmark  NRCT | *N=* 100.  Mean age (yrs): I: 74.5; C: 74.5.  % Male: 43.  Diagnosis: COPD.  Setting: Odense University Hospital, Svendborg.  Inclusion: COPD; exacerbation of COPD; > 40 yrs; >10 pack yrs; able to use a phone.  Exclusion: Communication problems; participation in other studies; systolic blood pressure < 100 mmHg; pH<7.35 or pO2 < 7.3 or saturation < 90 %; x-ray with lobar pneumonia or tumour or no X-ray taken; other serious diseases; cancer or severe HF; refused to participate; nurse strike, holiday, not possible to get a suitcase; death before discharge. | *N=*50. Post d/c telemedicine video consultations (TVC) with a nurse over one week period. Nurse made clinical observations, measured oxygen saturation levels and lung function; informed the pts how to prevent exacerbations and how to use the medication. TVC’c could be used 8am-3 pm every day and the pt could call telemedicine department in the same period of time using hotline for 28 day intervention period.   - Number of calls: At least 1. - Conducted: Within four week period. - Mean length of call: NR. - Person who made call: Nurse. - Content: NR. | *N=*50. NR. | Outcome measures:   - Readmission rates for COPD. - All-cause readmission rates. - Cumulative days of readmission.   Follow up: 28 days. | - No significant difference between I and C for number of pts readmitted for COPD (12% vs 22%) (-10% [-25;5]), or number of pts readmitted for all causes (16% vs 30%)( -14% [-30;2]) or cumulative days of readmission (2.0 vs 5.3 days) (-2 [-6;1]). |
| **Sorknaes *et al.*, 2013^23^**  Denmark  RCT | *N=*266  Mean age (yrs): I: 71 (10); C: 72 (9)  % Male: I: 40; C: 38  Diagnosis: AECOPD  Setting: 2 hospitals, Denmark.  Inclusion: COPD verified by spirometry (FEV1/FVC < 70%), admitted with AECOPD (defined by increased need for medicine and increased dyspnoea, increased expectorate volume or increased coughing), ≥40 years, and if residents of Funen.  Exclusion: able to use telephone and/or computer screen, in another scientific studies, BP < 100 mmHg, saturation < 90%, chest X-ray showing signs of malignancy or lobar pneumonia, diagnosed cancer or recurrence of cancer within last 5 yrs, septic shock, AMI or other serious medical conditions, CHF with ejection fraction <30%. | *N=* 132. In addition to UC, pts received daily teleconsultations by video. Conducted for 5 to 9 days starting within 24 hours of d/c. Pt made measurements and reported to nurse. Advice given: regular treatment, prevention of exacerbation, how to live with the disease and to empower pt.   - Number of calls: At least 1. - Conducted: In the week after teleconsultation. - Mean length of call: NR. - Person who made call: Nurse. - Content: NR. | *N=* 134. UC according to GOLD guidelines. Outpatient clinic consultation with a nurse 4 and 12 weeks. Nurse and pt made a plan for future course of action. | Outcome measures:   - Total hospital readmissions (COPD-related and non-COPD-related) - time before first readmission - hospital readmissions per patient   Follow up: 4, 8, 12 and 26 weeks | - No significant mean difference in total readmission between I and C (-0.08 [95% CI -0.25, 0.09] P-value= 0.35). - No significant mean difference in AECOPD readmission between I and C (-0.09 [95% CI -0.25, 0.07] P-value= 0.28). - No significant difference in time before first readmission and hospital readmissions per patient. |
